# TIMP-2 regulates 5-Fu resistance via the ERK/MAPK signaling pathway in colorectal cancer

**DOI:** 10.18632/aging.203793

**Published:** 2022-01-12

**Authors:** Guolin Zhang, Xin Luo, Zian Wang, Jianbin Xu, Wei Zhang, Engeng Chen, Qing Meng, Di Wang, Xuefeng Huang, Wei Zhou, Zhangfa Song

**Affiliations:** 1 Department of Colorectal Surgery, Sir Run Run Shaw Hospital of Zhejiang University, Hangzhou, China; 2 Department of Gastrointestinal Surgery, Shaoxing People’s Hospital, Shaoxing Hospital, Zhejiang University School of Medicine, Shaoxing, Zhejiang Province, China; 3 Department of Colorectal Surgery, Shaoxing People’s Hospital, Shaoxing Hospital, Zhejiang University School of Medicine, Shaoxing, Zhejiang Province, China; 4 Zhejiang Province Key Laboratory of Biological Treatment, Hangzhou, China

**Keywords:** TIMP-2, ERK/MAPK signaling pathway, 5-Fu, drug resistance, colorectal cancer

## Abstract

5-Fluorouracil (5-Fu) is the first-line chemotherapeutic option for colorectal cancer. However, its efficacy is inhibited by drug resistance. Cytokines play an important role in tumor drug resistance, even though their mechanisms are largely unknown. Using a cytokine array, we established that tissue inhibitor metalloproteinase 2 (TIMP-2) is highly expressed in 5-Fu resistant colorectal cancer patients. Analysis of samples from 84 patients showed that elevated TIMP-2 expression levels in colorectal patients were correlated with poor prognostic outcomes. In a 5-Fu-resistant patient-derived xenograft (PDX) model, TIMP-2 was also found to be highly expressed. We established an autocrine mechanism through which elevated TIMP-2 protein levels sustained colorectal cancer cell resistance to 5-Fu by constitutively activating the ERK/MAPK signaling pathway. Inhibition of TIMP-2 using an anti-TIMP-2 antibody or ERK/MAPK inhibition by U0126 suppressed TIMP-2 mediated 5-Fu-resistance in CRC patients. In conclusion, a novel TIMP-2-ERK/MAPK mediated 5-Fu resistance mechanism is involved in colorectal cancer. Therefore, targeting TIMP-2 or ERK/MAPK may provide a new strategy to overcome 5-Fu resistance in colorectal cancer chemotherapy.

## INTRODUCTION

Colorectal cancer (CRC), particularly advanced colorectal cancer, poses a significant challenge in clinical management and is associated with high mortality rates [1]. Moreover, the prognostic outcomes for patients with advanced CRC is poor [2]. 5-fluorouracil (5-Fu), which acts by interfering with cellular DNA synthesis and histone deacetylation, is recommended as a first-line chemotherapeutic option for CRC [3, 4]. Clinically, administration of 5-Fu combined with irinotecan or oxaliplatin is considered to be a relatively standard chemotherapeutic regimen [5]. Most patients show an initial effective response to 5-Fu, however, they later develop tumor progression, which is indicative of resistance [6–8]. The potential mechanisms of 5-Fu drug resistance have been reported [9–11]. However, specific molecular mechanisms of 5-Fu drug resistance have not been established.

Tumor resistance is closely associated with miRNAs dysregulation [12], promoter hypermethylation [13], and abnormal expressions of cell cycle-related proteins [14]. Due to the role of cytokines in physiological and pathological cell activities, studies are evaluating their potential roles in tumor drug resistance [15–19]. Cytokines are involved in drug metabolism, delivery, targeting and resistance [16, 20, 21].

As endoproteases, matrix metalloproteinases (MMPs) affect the integrity of extracellular matrix components [22]. Particularly, matrix metalloproteinase 2 (MMP-2) is associated with movement, migration, and metastasis of malignant cells [23–25]. Tissue inhibitor of matrix metalloproteinase 2 (TIMP-2) is a natural inhibitor of MMP-2 [26, 27]. TIMP-2 plays a dual role in cell physiology regulation. It promotes tumor growth via angiogenesis and, through apoptosis, it is also involved in inhibition of malignant cell proliferation [28–31]. TIMP-2 is also associated with tumor malignancy and resistance to chemotherapy in hepatoma, melanoma, and breast cancer [32–34]. In a previous study, Zhang et al. found that TIMP-2 siRNA effectively inhibited colorectal tumor cells (HCT116) invasion *in vitro* [35]. Clinical reports suggest that MMP-2 and TIMP-2 are more prevalent in CRC tissues than in normal tissues, with elevated expression levels in metastatic CRC compared to non-metastatic CRC [36–38]. Elevated TIMP-2 levels have particularly been reported in CRC patients with unfavourable chemotherapeutic responses [36]. However, it has not been established whether it has the same effects in all other tumors, and specific mechanisms of action have not been elucidated. Therefore, there is a need to evaluate the role of TIMP-2 in tumor cell resistance to 5-Fu therapy.

The ERK/MAPK signaling pathway is present in various types of cells. It is activated by dual phosphorylation of MAPKK kinase, catalyzed by the Thr-X-Tyr motif [39, 40]. Then, MAPKK is activated through the phosphorylation of MAPK kinase (MAPKKK) after which MAPKKK is activated by interactions with small GTPase and/or other proteases, thereby combining MAPK with cell surface receptors and with extracellular signals. Upon activation, MAPKs phosphorylate several protein kinases and transcription factors, including ERK1/2, JNK, p38MAPK, ERK5, NF-κB, and SOS [41, 42]. This signaling pathway regulates many critical physiological processes, such as cell growth, signal transduction, stress, and inflammatory responses [35, 43].

Various cytokines simultaneously activate ERK1/2 and ERK5, which then affect cell proliferation and differentiation [44–47]. Peng et al. found that the ERK1/2 signaling pathway plays an important regulatory role in CRC invasion and metastasis [48]. With regards to drug resistance in tumors, the ERK/MAPK signaling pathway plays an essential role in melanoma prognosis [49–51]. However, combined administration of BET and MEK inhibitors can significantly inhibit the growth of NRAS mutant melanoma and improve survival outcomes for cancer patients [50]. Moreover, these signaling pathways reactivate and play an important role in metastatic melanoma resistance to BRAF inhibition [51]. However, their roles in CRC 5-Fu resistance have not been established.

In this study, we investigated differences in cytokine expression profiles in serum samples of 5-Fu drug-resistant CRC patients. We found that 5-Fu resistant CRC patients exhibited elevated TIMP-2 levels, which were correlated with poor clinical prognoses. TIMP-2 was also found to be highly expressed in 5-Fu resistant CRC PDX models. Furthermore, TIMP-2 promoted CRC cell resistance to 5-Fu *in vitro*. Mechanistic analyses revealed that the ERK/MAPK signaling pathway is actively involved in 5-Fu resistance caused by TIMP-2, and its inhibitor, U0126, inhibits this resistance. From our findings, we hypothesized that TIMP-2 and the ERK/MAPK signaling pathway are excellent therapeutic targets for overcoming 5-Fu resistance in CRC.

## MATERIALS AND METHODS

### Antibodies and reagents

5-Fluorouracil (5-Fu) was obtained from MedChemExpress. Recombinant TIMP-2 was obtained from PeproTech (diluted to 10 ng/mL in the experiment). The antibody for TIMP-2 neutralization was obtained from R&D systems (diluted to 5 μg/mL in the experiment). Antibodies to MAPK (Erk1/2) (Cat No.4695), phospho-MAPK (Erk1/2) (Thr202/Tyr204) (Cat No.4370), Erk5 (Cat No.3552), phospho-Erk5 (Thr218/Tyr220) (Cat No.3371) and GAPDH (Cat No. 97166) were purchased from Cell Signaling Technology (CST). In the WB experiment, the above antibodies were diluted 1:1000. HRP-conjugated antibodies were obtained from Hangzhou Fude Biological Technology.

### Enzyme-linked immunosorbent assay (ELISA)

Cell culture supernatants or serum TIMP-2 levels were measured using a sandwich ELISA kit (Elabscience) according to the manufacturer’s instructions. Samples were assayed in triplicates.

### Ethical considerations

Ethical approval for this study was obtained from the ethical committee of Sir Run Run Shaw Hospital, School of Medicine, Zhejiang University (study number: 20140213-19). All animal experiments were in accordance with standard animal care guidelines.

### Study participants

Serum samples were obtained from CRC patients at the Key Laboratory of Biotherapy of Zhejiang province, Sir Run Run Shaw Hospital, School of Medicine, Zhejiang University. Samples were collected from 2008 to 2018. Serum was collected during chemotherapy after patients had been determined to be resistant to 5-Fu. To prevent cytokine decomposition, after extracting the serum from the blood, it was stored at −80°C. Two experienced pathologists analyzed cancer cell contents, histological tissue types, as well as tumor staging. 5-Fu based chemotherapy was administered to CRC patients, who were then operated on by senior surgeons. World Health Organization (WHO) approved indices for Overall survival (OS) and Disease-free survival (DFS) were used to evaluate treatment efficacies. A total of 84 patients were included in this study. Responses to 5-Fu were divided into two categories; 5-Fu sensitive and 5-Fu resistant CRC. Each group had 42 patients. This classification was based on tumor regression within six months following 5-Fu administration. During chemotherapy with 5-Fu-based chemotherapeutic drugs, if the patient is not checked for tumor progression, we identify these patients as sensitive to 5-Fu, otherwise the patient is considered to be resistant to 5-Fu. Regarding the PDX model, tumor cells were extracted from a 66-year-old male rectal cancer patient, who had been diagnosed with pathologic stage III adenocarcinoma. This patient was untreated and had received neither chemotherapy nor radiotherapy before surgery. Subsequent chemotherapy showed that the tumor was sensitive to 5-Fu.

### Cytokine array

Serum cytokine levels were determined by a protein cytokine array using the Human Cytokine Antibody Array-Membrane (ab193656), purchased from Abcam, Cambridge, UK. This technique is based on the principle of sandwich immunoassay. It comprises 120 coupled target anti-cytokines and the appropriate controls in duplicate. DLD-1 5-FuS and DLD-1 5-FuR cells were cultured in RPMI-1640 medium without fetal bovine serum and incubated at 37°C in a 5% CO_2_ environment for 24 h. Then, membranes were exposed to the chemiluminescence imaging system (LUMIPULSE G1200). Conditioned medium containing cytokines were evaluated according to manufacturer's protocols. Results were normalized using internal controls, and relative protein levels determined across four biological replicates.

### Cell cultures

Two CRC cell lines (DLD-1 cells and HCT116 cells) were obtained from the American Type Culture Collection (ATCC, Manassas). They were respectively cultured in RPMI-1640 (Genom) or Dulbecco's Modified Eagle Medium (DMEM) with higher glucose levels (Genom) containing 10% fetal bovine serum (GIBCO). Incubation was done at 37°C in a 5% CO_2_ atmosphere.

To generate 5-FU resistant cell lines, DLD-1 and HCT116 cells in the logarithmic growth phase were plated into a 6-well plate, at a density of 1×10^6^ cells per well. The starting 5-Fu concentration in the corresponding culture medium in each well was 0.1 μM. Incubation was done at 37°C in a 5% CO_2_ atmosphere for 2 days. Then, the cell culture medium was replaced with a culture medium that does not contain 5-Fu, and further incubated. Upon achievement of original cell growth rates, 5-Fu concentrations of the corresponding culture medium was adjusted to 2–3 times the original in each well. Further incubation was done for 2 days, after which the above experimental process was continued. CCK-8 was used to assess cell viability and to calculate the IC_50_ value. After about half a year, a tumor cell line that can survive normally at a stable concentration of 5-Fu was screened.

### Cell viability assay

Cell viability was determined using the Cell-Counting Kit-8 (CCK8) (Dojindo Molecular Technologies), following the manufacturers’ instructions. Absorbance was measured at 450 nm using a microplate reader. Experiments were performed in triplicates.

### RNA isolation and RT-qPCR

Total RNA was extracted from cells using the Trizol reagent (Invitrogen). cDNA was synthesized using the cDNA reverse transcriptase kit (Takara). LightCycler 480 real-time PCR system (Roche, Mannheim) was used to perform SYBR Green-based (Takara) quantitative real-time PCR (RT-qPCR). Glyceraldehyde-3-phosphate dehydrogenase (GAPDH) was used as the internal control. The 2−ΔΔCq relative quantification method was used to determine mRNA levels of target genes.

### siRNA interference

Small interfering RNA (siRNA) against TIMP-2 was obtained from Thermo Fisher Scientific. Transient transfection assays were performed using Lipofectamine 2000 (Thermo Fisher Scientific) following the manufacturers' instructions. Cellular drug resistance and cytokine secretion were analyzed by treating cells with 30 pg/ml TIMP-2 siRNA for two days.

### Western blot analysis

Cells were lysed using a RIPA lysis buffer (Solarbio Life Sciences). Protein concentrations were determined by Bicinchoninic acid assay (BCA, Beyotime Institute of Biotechnology). Proteins from each sample (25 μg) were separated by 10% SDS-PAGE (Beyotime Institute of Biotechnology) and transferred to polyvinylidene fluoride membranes (Immobilon-P). Membranes were blocked using 5% dried skimmed milk for 1 h at room temperature and incubated in the presence of primary antibodies at 4°C overnight. Subsequently, IgG conjugated goat anti-rabbit or IgG conjugated goat anti-mouse secondary antibodies were added and incubated for 1 h at room temperature. Blots were developed using an enhanced chemiluminescence detection reagent (Hangzhou Fude Biological Technology).

### Animal experiments

Four week old female BALB/c- nude mice from SiBeiFu Biotechnology Co., Ltd (Beijing) were used in this study. Briefly, tumor cells from CRC patients were subcutaneously implanted in the groins of nude mice. Then, mice were assigned into three groups of 6 mice each: Veh group (injection of saline), 5-FuS group (injection of 5-Fu but no drug resistance), 5-FuR group (injection of 5-Fu and develop resistance). The experimental group (5-FuS and 5-FuR group) was intraperitoneally administered with 5-Fu (30 mg/kg/dose) three times a week, while the Veh group was intraperitoneally administered with the same dose of saline. An initial reduction in tumor size in the experimental group followed by a re-growth of more than 2.0 cm diameter represented a successful establishment of a PDX model of colorectal tumor that is resistant to 5-Fu, which was defined as the 5-FuR group. After 5-Fu treatment, subcutaneous tumors of some mice continued to decrease in size, and this was defined as the 5-FuS group. Mice that were subcutaneously administered with saline as the control were the Veh group. At that time, the mice were injected with 5-Fu or saline about 12 times. Once the PDX model was obtained, blood samples were collected from eyelids of nude mice after which mice were sacrificed to obtain tumor tissues.

### Immunohistochemistry

Tumor tissue samples were fixed in 4% buffered paraformaldehyde solution, dehydrated and immersed in paraffin, then sliced into 4 μm thick sections. Epitope retrieval was performed by cooking the de-paraffinized sections under pressure in Tris-EDTA buffer (pH 9.0) for 20 min. Hydrogen peroxide (3%) in methanol solution was applied for 10 min to block endogenous peroxidase activity. Normal goat serum (10%) was then used to prevent non-specific binding for 30 min. Slides were incubated for 1 h at 4°C in TIMP-2 antibody solution diluted at 1:20 followed by incubation with a secondary antibody for 30 min at room temperature. Then, sections were developed using a DAB kit (Shanghai Gene Co., Ltd) and counterstained with hematoxylin (Sigma). For semi-quantitative assay of IHC staining, staining intensity was scored from 0 to 4 (0, absent; 1, weak; 2, moderate; 3, intense; 4, extremely intense). Final IHC score for each sample was determined by three independent senior pathologists. By observing multiple visual fields, each pathologist gave two average scores.

### Statistical analysis

Data from three independent experiments tested in triplicates are presented as means ± SD. Data were analyzed using SPSS (version 22.0), Image J (version 2.0) and GraphPad Prism (version 7.0) software. A Combination index (CI) of 1.0 indicated an additive effect, while CI<1 suggested synergy. Alternative CI values indicated antagonism. Experimental data were examined for consistency to a normal distribution using the one-sample Kolmogorov-Smirnov test. An independent sample *t*-test or non-parametric test was used to analyze the experimental results. Comparisons between survival curves were tested for statistical significance using either a Log-rank test or COX regression analysis. In all cases, *p* values were two-sided. *p* ≤ 0.05 was considered significant.

### Ethical approval and consent to participate

This study was approved by the local ethics committee of Sir Run Run Shaw Hospital, School of Medicine, Zhejiang University (study number: 20140213-19). All animal experiments were in accordance with standard animal care guidelines.

### Availability of data and materials

The data supporting these findings are available from the Department of colorectal surgery, Sir Run Run Shaw Hospital of Zhejiang University but restrictions apply to the availability of these data, which were used under license for the current study, and are therefore, not publicly available. Data are, however, available from the authors upon reasonable request and with permission from the Department of colorectal surgery, Sir Run Run Shaw Hospital of Zhejiang University.

## RESULTS

### TIMP-2 was elevated in 5-FU resistant CRC patients and correlated with poor prognosis

**Figure 1:**
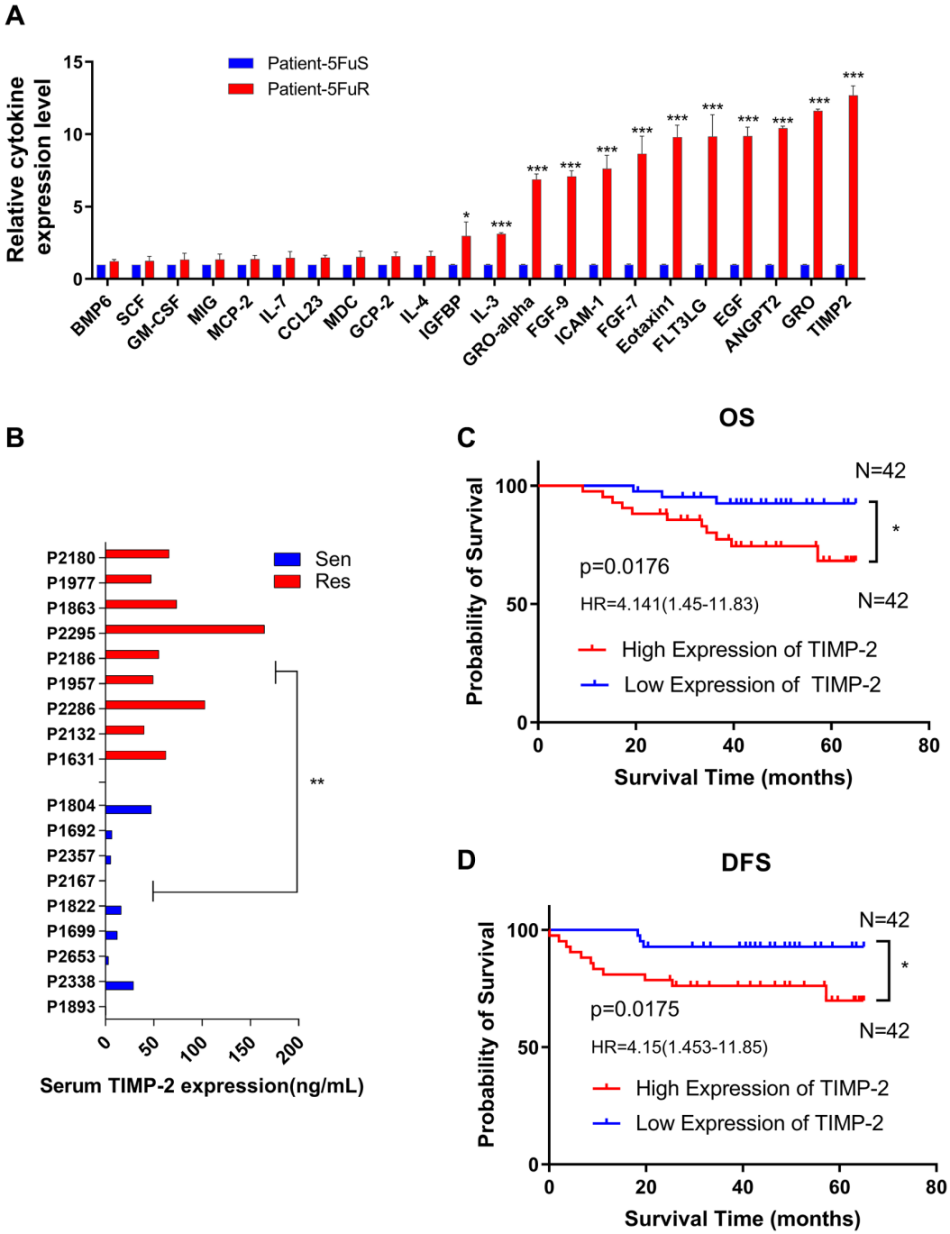
**TIMP-2 is elevated in 5-Fu resistant CRC patients and predicts clinical outcomes.** (**A**) Relative cytokine expression levels in the serum of 5-Fu sensitive and resistant patients. Patient details are shown in Table 1. Sen, sensitive patients. Res, resistant patients. (**B**) Differences in TIMP-2 protein expression levels in non-resistant (*n* = 9) and resistant patients (*n* = 9) with colorectal cancer. Patient details are shown in Table 2. Sen, sensitive patients. Res, resistant patients. (**C**) 6-year OS Kaplan–Meier survival curves for 84 colorectal cancer patients, differential grouping based on TIMP-2 expression (36.6 ng/ml) in serum. Table 3 shows patient information. (**D**) 6-year DFS Kaplan–Meier survival curves for 84 colorectal cancer patients, differential grouping based on TIMP-2 expression (36.6 ng/ml) in serum. Table 3 shows patient information. (**A**, **B**) ^*^p < 0.05, ^**^p < 0.01, ^***^p < 0.001 by unpaired Student’s *t*-test. (**C**, **D**) ^*^p < 0.05 by logrank (Mantel-Cox), HRs are shown in the figures.

Cytokines are important in drug resistance. First, we clinically selected three typical 5-Fu-resistant and three typical 5-Fu-sensitive CRC patients. Patient characteristics are shown in Table 1. In this study, cytokines such as TIMP-2, GRO, ANGPT2, and EGF, among others, were found to be significantly elevated in serum from 5-Fu-resistant patients (Figure 1A). Since TIMP-2 exhibited the greatest change in expression levels, we hypothesized that TIMP-2 is the key cause of CRC resistance to 5-Fu. To validate our hypothesis, we evaluated TIMP-2 serum levels in nine 5-Fu-resistant and nine 5-Fu-sensitive CRC patients using ELISA. Serum TIMP-2 protein levels in 5-Fu resistant CRC patients was 73.61 ng/ml, 5.3 times higher than those of 5-Fu sensitive CRC patients (13.57 ng/ml) (Figure 1B). Patient characteristics are shown in Table 2. Prognostic outcomes for clinical patients are of great concern to oncologists. Therefore, we evaluated protein levels of TIMP-2 in serum of 84 CRC patients undergoing 5-Fu-based chemotherapy and correlated it with prognosis. Characteristics of these patients are shown in Table 3. Median follow-up time was 54.4 months. Using the median value (36.60 ng/ml) of TIMP-2 protein expression level as the cut-off, patients were assigned into two groups: TIMP-2 high expression group (*n* = 42) and TIMP-2 low expression group (*n* = 42). According to major clinical outcomes of Overall survival (OS) and Disease-free survival (DFS), TIMP-2 high expression group exhibited worse prognostic outcomes, relative to TIMP-2 low expression group (Figure 1C and 1D).

**Table 1:** Characteristics of patients involved in cytokine screening.

**Patient**	**Age (years)**	**Sex**	**Stage**	**Histology**	**Chemotherapy**
**P0221**	78	M	IVA	Adenocarcinoma	5-Fu + Oxaliplatin + Bevacizumab
**P0258^#^**	69	F	IIIB	Adenocarcinoma	5-Fu + Oxaliplatin + Bevacizumab
**P0378^#^**	57	F	IVA	Mucus adenocarcinoma	5-Fu + Oxaliplatin + Bevacizumab
**P0855**	64	M	IIIC	Adenocarcinoma	5-Fu + Irinotecan + Oxaliplatin + Cetuximab
**P1061**	60	F	IIIB	Adenocarcinoma	5-Fu + Oxaliplatin + Bevacizumab
**P1392^#^**	77	M	IIIC	Adenocarcinoma	5-Fu+Irinotecan + Oxaliplatin + Cetuximab

^#^Refers to colorectal cancer patients resistant to 5-Fu.

**Table 2:** Characteristics of patients involved in assessment of 5-Fu sensitivity or resistance.

**Patient**	**Age (years)**	**Sex**	**Stage**	**Histology**	**Chemotherapy**
**P1631^#^**	66	M	IVA	Adenocarcinoma	5-Fu + Irinotecan + Oxaliplatin + Bevacizumab
**P1692**	61	F	IVA	Adenocarcinoma	5-Fu + Irinotecan + Oxaliplatin + Bevacizumab
**P1699**	78	F	IVA	Mucus adenocarcinoma	5-Fu + Oxaliplatin + Bevacizumab
**P1804**	21	F	IIIC	Mucus adenocarcinoma	5-Fu + Irinotecan + Oxaliplatin
**P1822**	76	M	IVB	Adenocarcinoma	5-Fu + Irinotecan + Oxaliplatin + Bevacizumab
**P1863^#^**	47	F	IVB	Mucus adenocarcinoma	5-Fu + Irinotecan + Oxaliplatin + Bevacizumab
**P1893**	55	M	IVA	Adenocarcinoma	5-Fu + Oxaliplatin
**P1957^#^**	55	M	IVB	Adenocarcinoma	5-Fu + Irinotecan + Oxaliplatin + Cetuximab
**P1977^#^**	51	F	IVB	Adenocarcinoma	5-Fu + Irinotecan + Oxaliplatin + Bevacizumab
**P2132^#^**	55	M	IVB	Adenocarcinoma	5-Fu + Irinotecan + Oxaliplatin + Bevacizumab
**P2167**	66	M	IVA	Adenocarcinoma	5-Fu + Irinotecan + Oxaliplatin + Bevacizumab
**P2180^#^**	53	F	IVB	Adenocarcinoma	5-Fu + Oxaliplatin
**P2186^#^**	65	M	IIIC	Adenocarcinoma	5-Fu + Irinotecan + Oxaliplatin
**P2286^#^**	59	M	IVA	Adenocarcinoma	5-Fu + Irinotecan + Oxaliplatin + Cetuximab + Bevacizumab
**P2295^#^**	48	M	IIIC	Adenocarcinoma	5-Fu + Irinotecan + Oxaliplatin + Bevacizumab
**P2338**	66	M	IVB	Adenocarcinoma	5-Fu + Irinotecan + Oxaliplatin + Cetuximab
**P2357**	70	M	IIIB	Adenocarcinoma	5-Fu + Irinotecan + Bevacizumab
**P2653**	65	M	IIIC	Adenocarcinoma	5-Fu + Irinotecan + Oxaliplatin + Cetuximab

^#^Refers to colorectal cancer patients resistant to 5-Fu.

**Table 3:** Correlations between patient serum TIMP-2 levels and clinical characteristics.

**Characteristics**	**Total**	**serum TIMP-2 levels**		**OR**	**95% CI**	***p*-value**
		**<36.6 ng/ml**	**≥36.6 ng/ml**			
**All Cases**	84	42 (39.0%)	42 (61.0%)			
**Age (years)**						
≥65	45	19 (42.2%)	26 (57.8%)			
<65	39	23 (59.0%)	16 (41.0%)	0.508	0.213–1.214	0.189
**Gender**						
Male	48	19 (39.6%)	29 (60.4%)			
Female	36	23 (63.9%)	13 (36.1%)	0.37	0.151–1.043	0.179
**Stage**						
IIIA	7	3 (42.9%)	4 (57.1%)			
IIIB	62	33 (53.2%)	29 (46.8%)			
IIIC	10	6 (60.0%)	4 (40.4%)			
IVA	3	0	3 (100%)			
IVB	2	0	2 (100%)			0.215
**Histological type**						
Adenocarcinoma	67	33 (49.3%)	34 (50.7%)			
Mucus adenocarcinoma	14	8 (57.1%)	6 (42.9%)			
Others	3	1 (33.3%)	2 (66.7%)			0.728

*p*-value calculated by the Chi-square test.

### TIMP-2 levels were upregulated in 5-Fu resistant CRC cells and in PDX models

**Figure 2:**
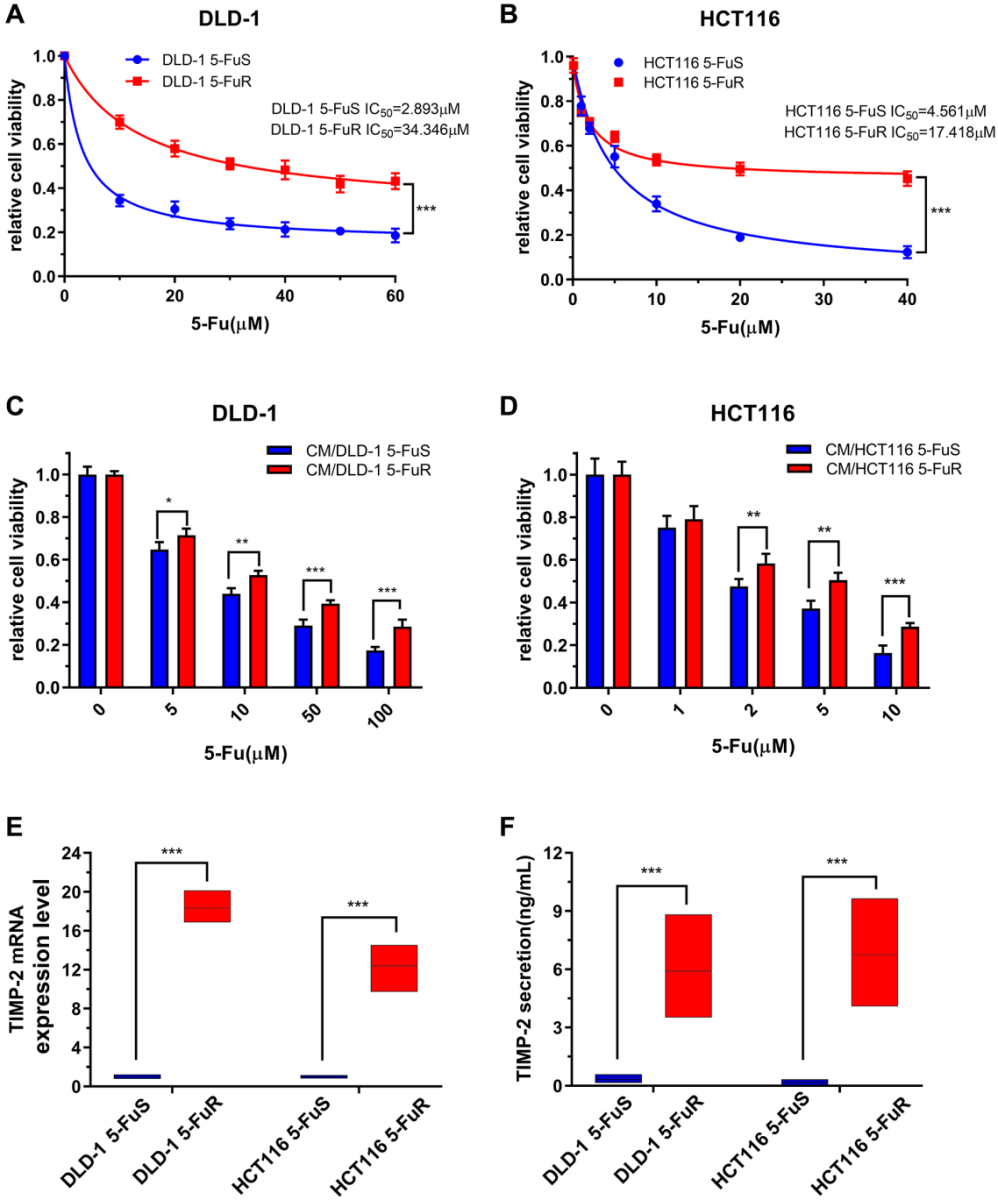
**Upregulation of TIMP-2 in 5-Fu resistant CRC cells *in vitro***. (**A**, **B**) Relative cell viabilities of DLD-1 5-FuS cells and DLD-1 5-FuR cells, HCT116 5-FuS cells and HCT116 5-FuR cells under increasing concentrations of 5-Fu for 3 days. (**C**, **D**) Relative cell viabilities of DLD-1 5-FuS cells and HCT116 5-FuS cells in increasing concentrations of 5-Fu for 3 days after culture in conditioned medium of DLD-1 5-FuR cells or HCT116 5-FuR cells for 2 days. (**E**) mRNA expression levels of TIMP-2 in paired DLD-1 5-FuS cells and DLD-1 5-FuR cells, HCT116 5-FuS cells and HCT116 5-FuR cells. (**F**) Differences in TIMP-2 protein expression levels in paired DLD-1 5-FuS cells and DLD-1 5-FuR cells, HCT116 5-FuS cells and HCT116 5-FuR cells. Data from triplicate wells in 3 independent experiments. (**A**, **B**) ^***^p < 0.001 by 2 way ANOVA test. (**C**–**F**) ^*^p < 0.05, ^**^p < 0.01, ^***^p < 0.001 by Student’s *t*-test.

By gradually increasing 5-Fu concentrations in the culture medium, we developed resistant cell lines from two CRC cell lines, DLD-1 and HCT116 [52]. These were named DLD-1 5-FuR and HCT116 5-FuR, respectively, while primary cells lines were named DLD-1 5-FuS and HCT116 5-FuS. Cell activity data obtained at different concentrations were used to determine the 50% inhibitory concentration (IC_50_). IC_50_ value for 5-Fu was 11.8-fold in DLD-1 5-FuR, compared to DLD-1 5-FuS. In HCT116 5-FuR and HCT116 5-FuS, IC_50_ was 3.81-fold (Figure 2A and 2B). Given the relationship between cytokines and tumor resistance [15, 53], we designed a cell culture medium (CM) exchange experiment to verify the effects of cytokines on tumor cell resistance. We used the DLD-1 5-FuR cell culture medium to culture DLD-1 5-FuS cells. These experiments showed that DLD-1 5-FuS cells co-cultured in DLD-1 5-FuR medium were more tolerant to different 5-Fu concentrations than those cultured in the conditioned medium (Figure 2C). The same experiment was repeated in HCT116 5-FuS cells, and similar results were obtained (Figure 2D). It was found that 5-Fu-resistant cell lines secrete cytokines that cause drug resistance.

To determine whether TIMP-2 protein is involved in drug-resistance, we assessed the expression levels of TIMP-2 by real-time quantitative PCR and ELISA. Semi-quantitative mRNA analysis showed that TIMP-2 transcription levels in drug-resistant cell lines were significantly higher than those of sensitive cell lines (Figure 2E). Assessment of TIMP-2 protein levels by ELISA showed that it was highly secreted in drug-resistant cell lines, including DLD-1 5-FuR and HCT116 5-FuR (Figure 2F).

**Figure 3:**
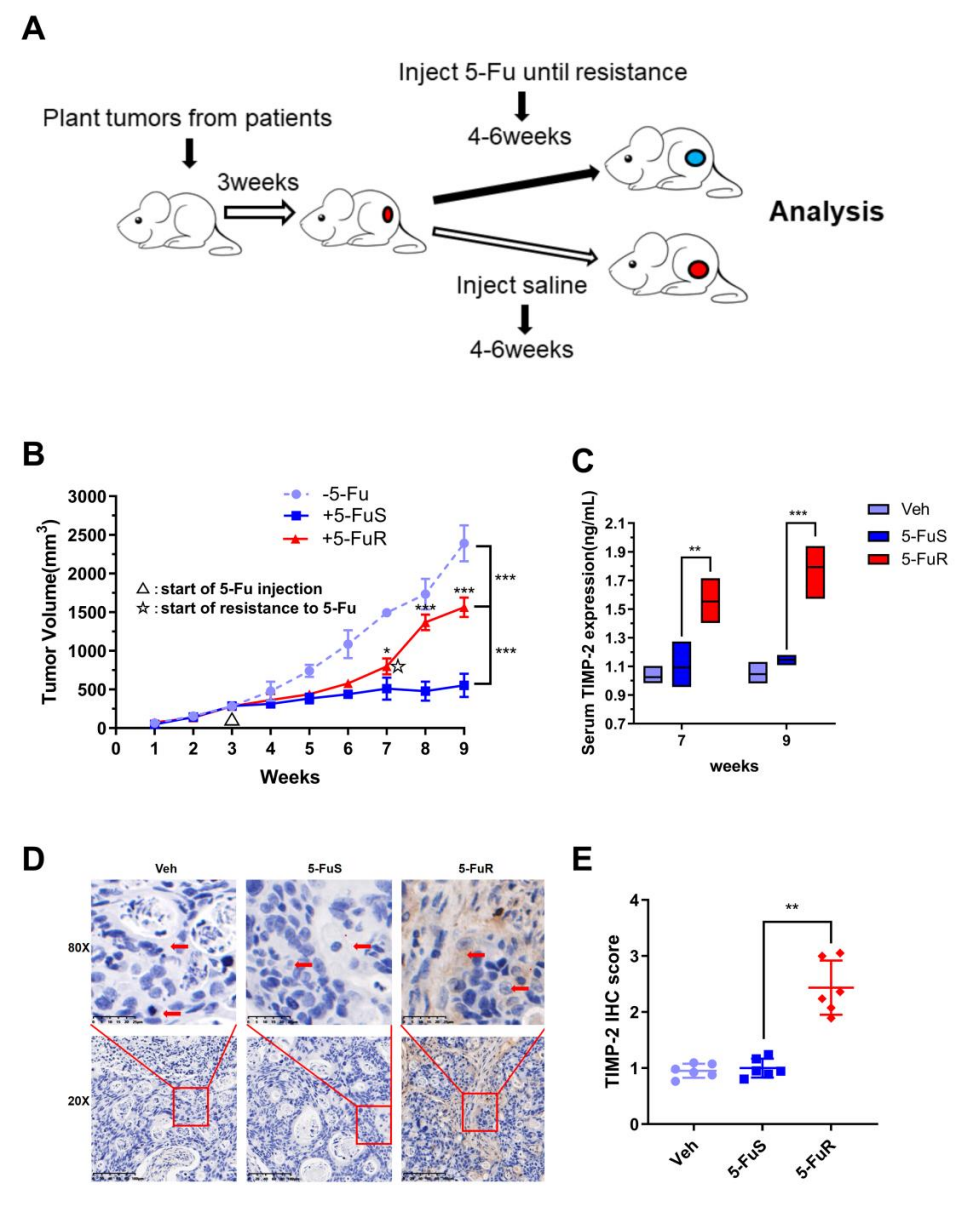
**Activation of TIMP-2 in 5-Fu resistant PDX models of CRC *in vivo.*** (**A**) Schematic presentation of the constructing of a PDX-drug resistance model. (**B**) Changes in tumor volumes for Veh, 5-FuS and 5-FuR group PDX mice models during the experiment. (**C**) Differences in TIMP-2 protein levels in Veh, 5-FuS and 5-FuR group PDX mice models. (**D**) IHC for typical TIMP-2 staining images of subcutaneous tumors formed in Veh, 5-FuS and 5-FuR group PDX mice models. (**E**) Semi-quantitative IHC staining scores for TIMP-2 as shown in Figure 3C. Data is presented as mean ± SD. Three mice and 6 tumors per experimental group. ^*^p < 0.05, ^**^p < 0.01, ^***^p < 0.001 by Student’s *t*-test or two-way ANOVA.

To further show that TIMP-2 was also up-regulated during 5-Fu treatment *in vivo*, we used patient-derived xenograft (PDX) models. The PDX model maintains the donor's original biological behaviors and molecular characteristics [54–57]. Following the necessary construction processes, we constructed a PDX model of colorectal tumor with resistance to 5-Fu (Figure 3A). When the experimental group was treated with 5-Fu, subcutaneous tumors in the experimental group (5-FuS and 5-FuR group) began to be under control. After about 4 weeks of treatment, subcutaneous tumors of 5-FuS group PDX mice began to be resistant to 5-Fu, implying that the PDX model of colorectal tumor with resistance to 5-Fu had successfully been constructed (Figure 3B). TIMP-2 protein levels in the serum of 5-Fu-resistant PDX models were found to be significantly higher than those of sensitive strains (Figure 3C). Immunohistochemical (IHC) analysis showed that tumor tissues, which showed elevated TIMP-2 expression levels exhibited resistance to 5-Fu (Figure 3D). Semi-quantitative immunohistochemical analysis further affirmed these results (Figure 3E). Similar results were obtained from tested patient serum.

### TIMP-2 promotes CRC cell resistance to 5-Fu through an autocrine mechanism

**Figure 4:**
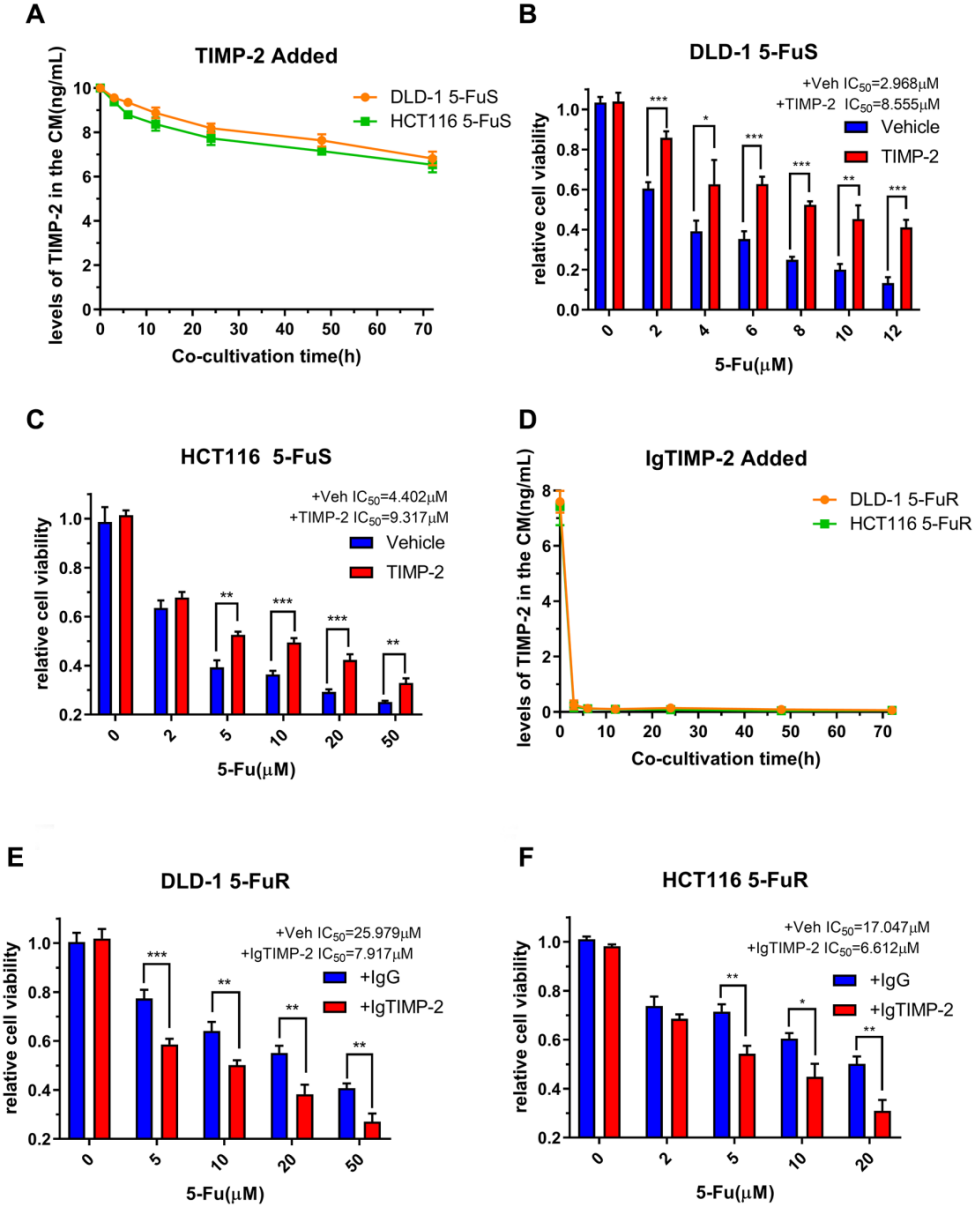
**TIMP-2 promotes CRC cell resistance to 5-Fu through an autocrine mechanism.** (**A**) Changes in TIMP-2 protein levels in the cell culture medium (CM) of DLD-1 5-FuS cells and HCT116 5-FuS cells 3 days after treatment with 10 ng/mL of recombinant TIMP-2. (**B**, **C**) Relative cell viabilities of DLD-1 5-FuS cells and HCT116 5-FuS cells under increasing concentrations of 5-Fu for 3 days after culture with 10 ng/mL of recombinant TIMP-2. (**D**) Changes in TIMP-2 protein levels in the cell culture medium (CM) of DLD-1 5-FuR cells and HCT116 5-FuR cells for 3 days of culture with 5 μg/mL of TIMP-2 neutralizing antibody. (**E**, **F**) Relative cell viabilities of DLD-1 5-FuR cells and HCT116 5-FuR cells under increasing concentrations of 5-Fu for 3 days of culture with control IgG or 5 μg/mL of TIMP-2 neutralizing antibody. Data from triplicate wells for 3 independent experiments. ^*^p < 0.05, ^**^p < 0.01, ^***^p < 0.001 by Student’s *t*-test or one-way ANOVA.

We have confirmed that TIMP-2 is elevated in 5-FU resistant CRC patients and is correlated with poor prognostic outcomes. Furthermore, we confirmed that TIMP-2 is closely associated with 5-Fu resistance in CRC cells. Next, we set to confirm that it is TIMP-2 and not other cytokines that cause 5-Fu resistance. This assay was done by adding recombinant TIMP-2 to the culture medium of 5-Fu sensitive cell lines and adding the neutralization TIMP-2 antibody to the culture medium of 5-Fu resistant cell lines. Following treatment of CRC cell lines (DLD-1 5-FuS and HCT116 5-FuS) with recombinant TIMP-2, changes in TIMP-2 protein levels in cell culture medium (CM) of DLD-1 5-FuS cells and HCT116 5-FuS cells were determined. TIMP-2 protein levels were elevated for 3 days, comparable to levels in the culture medium of 5-Fu resistant cell lines (Figure 4A). Interestingly, after the addition of TIMP-2, less sensitivity to 5-Fu and increased IC_50_ was observed in the 5-Fu sensitive cell lines (Figure 4B and 4C). When the TIMP-2 neutralization antibody was added to the culture medium of DLD-1 5-FuR and HCT116 5-FuR cells, TIMP-2 protein levels were significantly suppressed (Figure 4D). Consistent with our prediction, IC_50_ of both 5-Fu resistant cell lines were significantly decreased, indicative of increased sensitivity of cells to drugs (Figure 4E and 4F).

**Figure 5:**
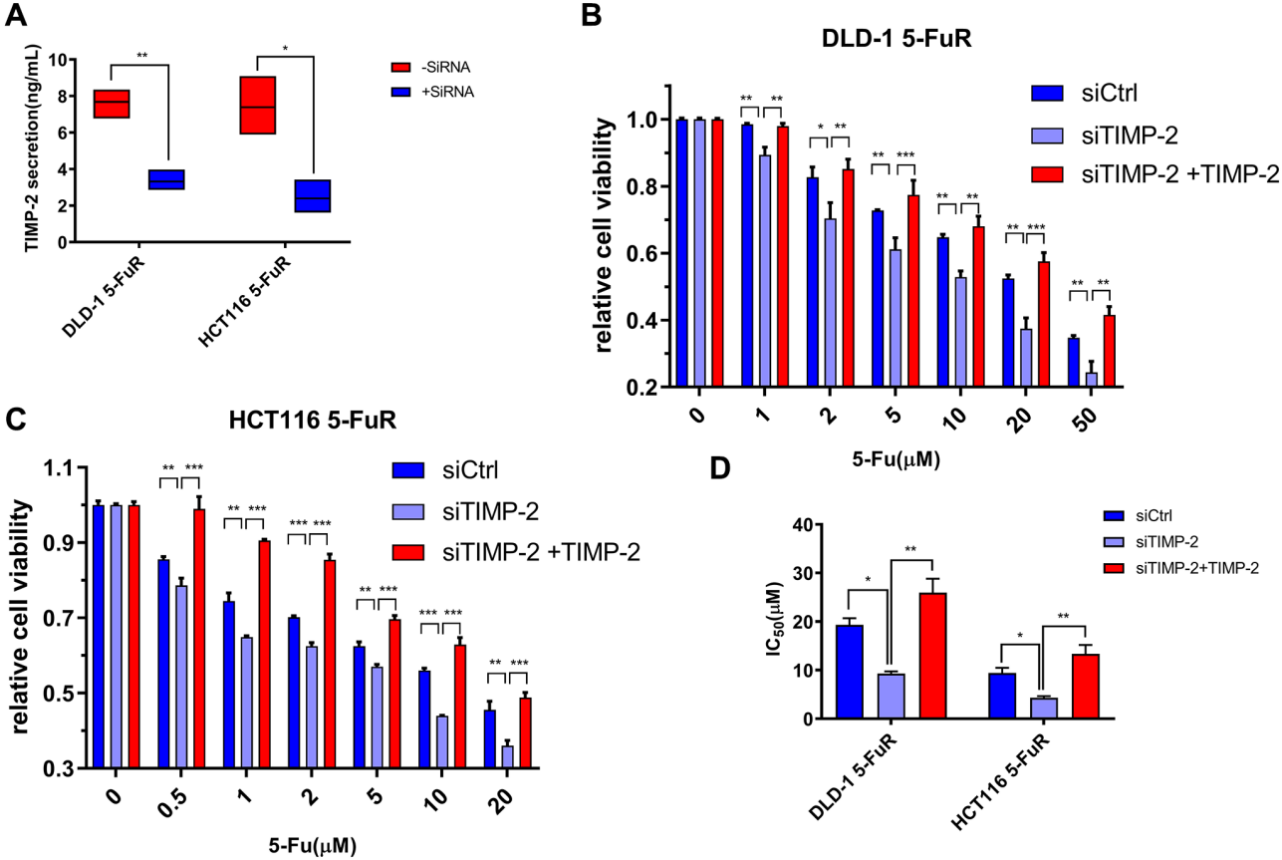
**Knockdown of TIMP-2 overcomes 5-Fu resistance in CRC cells.** (**A**) Changes in expression levels of TIMP-2 in DLD-1 5-FuR cells and HCT116 5-FuR cells after control siRNA or TIMP-2 siRNA (30 pg/ml) transfections. (**B**, **C**) Relative cell viabilities of DLD-1 5-FuR cells and HCT116 5-FuR cells under increasing concentrations of 5-Fu for 3 days of culture with TIMP-2 siRNA (30 pg/ml) or TIMP-2 siRNA (30 pg/ml) and recombinant TIMP-2 (10 ng/ml) together. (**D**) Differences in 5-Fu concentrations for 50% inhibition of cell growth (IC_50_) between the six groups of cells in Figure 5B and 5C above. Data from triplicate wells for 3 independent experiments. ^*^p < 0.05, ^**^p < 0.01, ^***^p < 0.001 by Student’s *t*-test or one-way ANOVA.

To validate the relationship between 5-Fu resistance and TIMP-2 protein expression levels in colorectal tumors, we used small interfering RNA (siRNA) to knock down TIMP-2 expression in cell lines. siRNA against TIMP-2 showed excellent knock-down efficiency in DLD-1 5-FuR and HCT116 5-FuR cells (Figure 5A). Besides, DLD-1 5-FuR and HCT116 5-FuR cells regained sensitivity to 5-Fu after knock-down of TIMP-2 expression by siRNA (Figure 5B and 5C). Remarkably, the higher the concentration of 5-Fu in the culture solution, the more apparent the above effect. Addition of recombinant TIMP-2 protein to the siRNA-treated DLD-1 5-FuR and HCT116 5-FuR cells restored the resistance of cell lines to 5-Fu (Figure 5B and 5C). The IC_50_ for each group of cells in the above experiment are shown in Figure 5D. These results show that TIMP-2 induces 5-Fu resistance in CRC cells.

### TIMP-2 induces 5-Fu resistance by activating ERK/MAPK in CRC cells

We further determined the signaling pathway involved in TIMP-2 induced 5-Fu resistance in CRC. It has been reported that TIMP-2 mediates endothelial proliferation, formation of a capillary tube in obesity, and promotes tumor invasion in advanced squamous cell carcinomas [58, 59] by activating the ERK/MAPK signaling pathway. The role of the ERK/MAPK signaling pathway in tumor resistance has been widely reported [60–62]. Therefore, we explored the underlying mechanisms through which TIMP-2 mediates drug resistance by analyzing the expression levels of key proteins in the ERK/MAPK signaling pathway.

**Figure 6:**
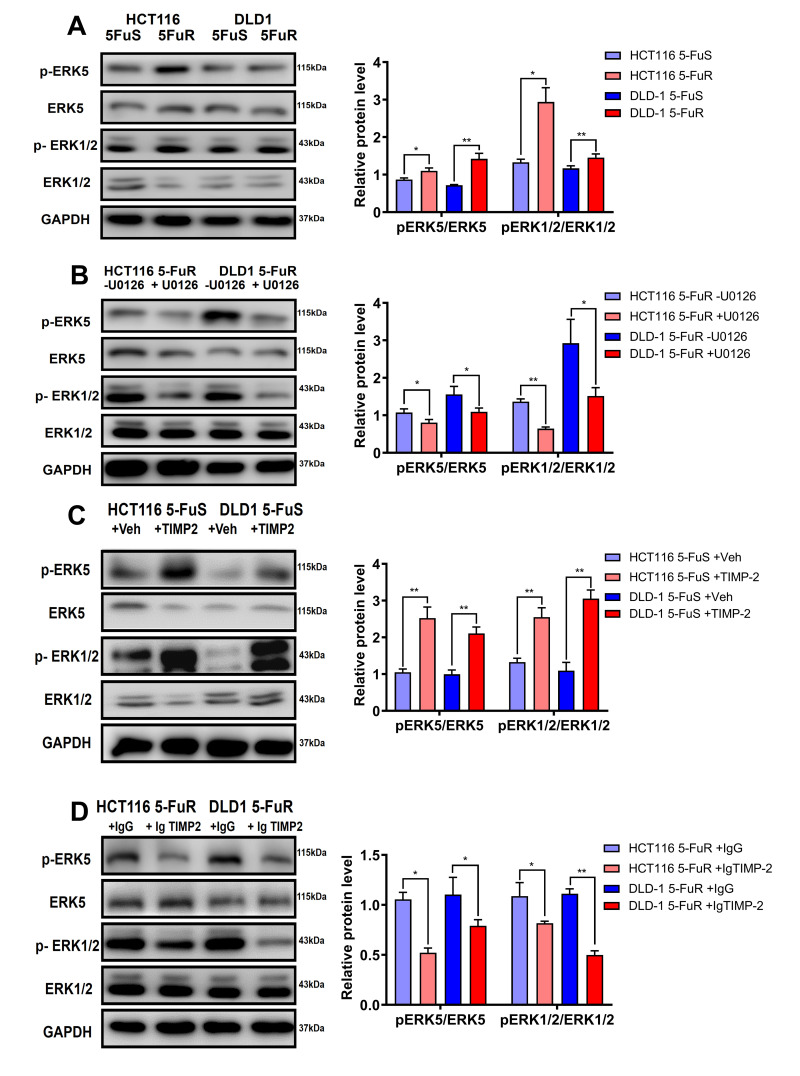
**TIMP-2 sustains the activation of ERK/MAPK in CRC cells.** (**A**) Immunoblotting of phosphorylated ERK1/2 and ERK5 in DLD-1 5-FuS cells and DLD-1 5-FuR cells, HCT116 5-FuS cells and HCT116 5-FuR cells. (**B**) Immunoblotting of phosphorylated ERK1/2 and ERK5 in DLD-1 5-FuR cells and HCT116 5-FuR cells cultured with 5 μM of U0126 for 2 days, which down-regulates ERK/MAPK signaling. (**C**) Immunoblotting of phosphorylated ERK1/2 and ERK5 in DLD-1 5-FuS cells and HCT116 5-FuS cells cultured with 10 ng/mL of recombinant TIMP-2 for 6 h. (**D**) Immunoblotting of phosphorylated ERK1/2 and ERK5 in DLD-1 5-FuR cells and HCT116 5-FuR cells cultured with control IgG or 5 μg/mL of TIMP-2 neutralizing antibody for 6 h. Band intensities of western blotting for p-ERK5/ ERK5 and p-ERK1/2/ERK1/2 were analyzed. ^*^p < 0.05, ^**^p < 0.01, ^***^p < 0.001 by Student’s *t*-test.

When compared to DLD-1 5-FuS and HCT116 5-FuS cells, levels of p-ERK1/2/ERK1/2 and p-ERK5/ERK5 were found to be significantly elevated in DLD-1 5-FuR and HCT116 5-FuR cells, implying that activation of ERK1/2 was accompanied by ERK5 phosphorylation (Figure 6A). Since Erk1/2 and pErk1/2 antibodies can recognize Thr202 and Tyr204 sites of Erk1 and Thr185 as well as Tyr187 sites of Erk2, double bands are shown in the Figure 6.

To confirm the effects of the ERK/MAPK signaling pathway on drug resistance in CRC, we performed a series of experiments. U0126 is an ERK/MAPK signaling pathway inhibitor. Phosphorylation of ERK1/2 and ERK5 in both DLD-1 5-FuR and HCT116 5-FuR cells were markedly inhibited by U0126 treatment (Figure 6B). Moreover, we evaluated the role of TIMP-2 in activation of the ERK/MAPK signaling pathway in CRC cells. Recombinant TIMP-2 treatment significantly enhanced ERK1/2 and ERK5 phosphorylation in both DLD-1 5-FuS and HCT116 5-FuS cells (Figure 6C). However, addition of TIMP-2 neutralization antibody resulted in significantly decreased phosphorylation levels of ERK1/2 and ERK5 in both resistant cell lines (Figure 6D).

### U0126 inhibits 5-Fu resistance in CRC through the ERK/MAPK signaling pathway

**Figure 7:**
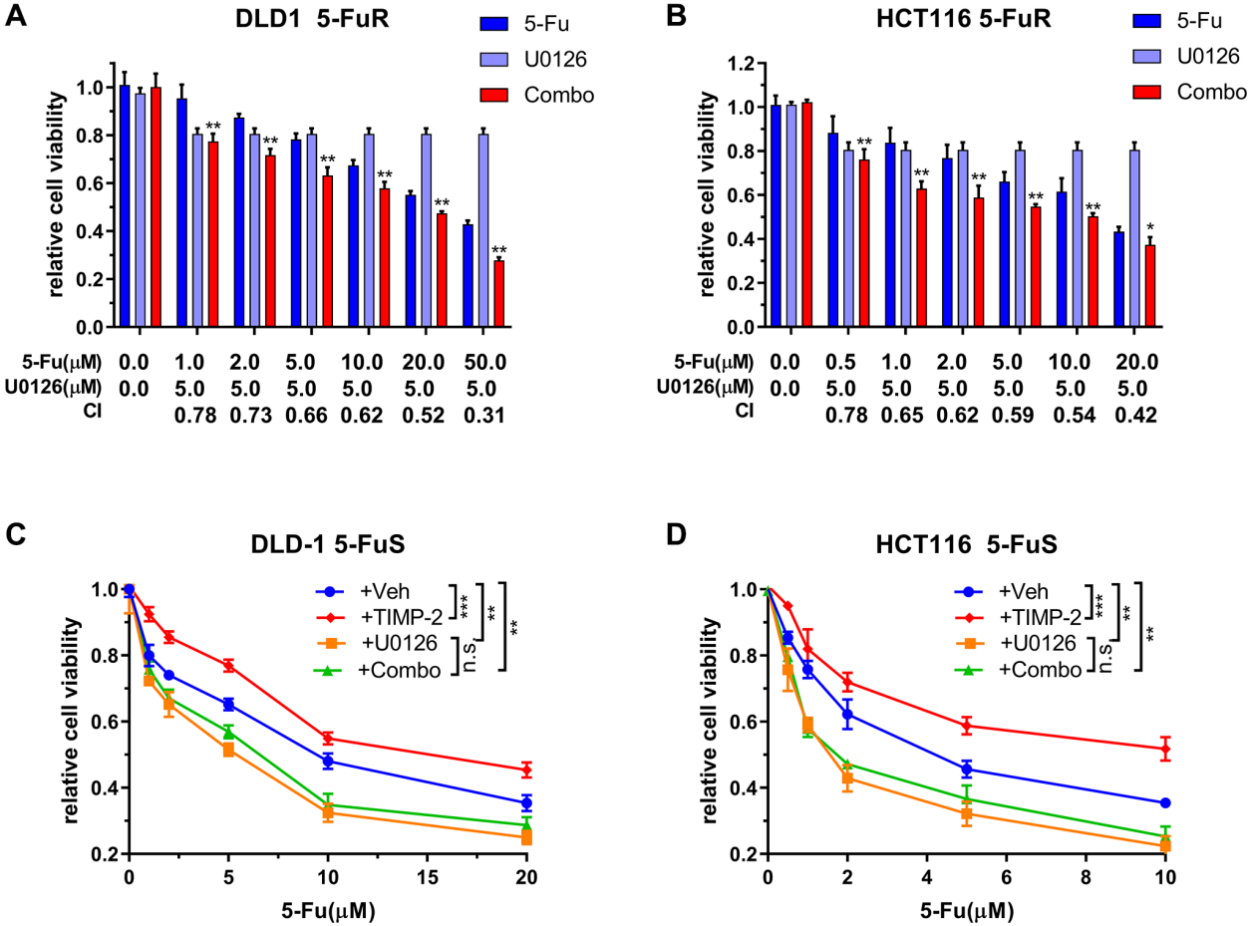
**U0126 inhibits 5-Fu resistance in CRC through the ERK/MAPK signaling pathway.** (**A**, **B**) Synergistic effects of U0126 and 5-Fu on DLD-1 5-FuR and HCT116 5-FuR cells. Combo = 5-Fu + U0126. (**C**, **D**) Knockdown ERK/MAPK by U0126 blocks TIMP-2 induced 5-Fu resistance in CRC cells. DLD-1 5-FuS and HCT116 5-FuS cells were cultured with 5 μM of U0126 for 24 h and then cultured with recombinant TIMP-2 (10 ng/ml) for 6 h, followed by increasing concentrations of 5-Fu treatment for 3 days. Combo = TIMP-2 + U0126. Combination index (CI) is presented below the bars. Data from triplicate wells of 3 independent experiments. (**A**, **B**) ^*^p < 0.05, ^**^p < 0.01 by Student’s *t*-test between group +5Fu and group Combo. (**C**, **D**) ^*^p < 0.05, ^**^p < 0.01, ^***^p < 0.001 by one-way ANOVA or two-way ANOVA.

We have shown that the ERK/MAPK signaling pathway is vital for 5-Fu resistance in CRC, therefore, we determined whether U0126 can inhibit the drug resistance process. Synergistic effects were used to analyze the impact of U0126 on the ERK/MAPK signaling pathway in CRC cell resistance. Treatment of CRC resistant cell lines using different concentrations of 5-Fu and U0126, alone or in combination, exhibited different effects. Therefore, we calculated the Combination index (CI) values, which we used to quantitatively determine interactions between the two drugs, for evaluating the combined effects of U0126 and 5-Fu. There was a strong synergistic effect from the combined U0126 and 5-Fu on both DLD-1 5-FuR and HCT116 5-FuR cells (Figure 7A and 7B).

To further determine that U0126 can reverse TIMP-2-induced 5-Fu resistance in CRC through the ERK/MAPK signaling pathway, we downregulated ERK/MAPK in DLD-1 5-FuS and HCT116 5-FuS cells using U0126. Then, cells were treated with recombinant TIMP-2, followed by increasing the concentrations of 5-Fu in the culture (Figure 7C and 7D). Even though the addition of recombinant TIMP-2 induced resistance to 5-Fu in DLD-1 5-FuS and HCT116 5-FuS cells containing ERK/MAPK, TIMP-2 did not induce 5-Fu resistance in CRC cells with downregulated ERK/MAPK signaling pathway (Figure 7C and 7D). These findings suggest that the ERK/MAPK signaling pathway plays a pivotal role in TIMP-2 mediated resistance of colorectal cancer cells to 5-Fu. When the ERK/MAPK signaling pathway is blocked, TIMP-2 induced 5-Fu resistance is significantly inhibited. The inhibitor of the ERK/MAPK signaling pathway, U0126, effectively inhibited 5-Fu resistance in colorectal cancer cells. This finding provides a basis for future development of small molecule drugs that antagonize 5-Fu resistance in tumors.

## DISCUSSION

In recent years, global incidences of CRC have gradually increased, especially among the elderly. Advanced CRC has been attributed to 5-Fu resistance. Cytokines in the para cancerous and circulating systems affect immune responses, occurrence, and metastasis of tumors as well as tumor drug resistance [15–18]. We found that TIMP-2 serum levels in 5-Fu resistant CRC patients were elevated.

TIMP-2 belongs to the tissue inhibitor of metalloproteinase (TIMP) family. This gene family encodes natural inhibitors of matrix metalloproteinases (MMPs), a group of peptidases involved in degradation of the extracellular matrix (ECM). Moreover, encoded proteins have a unique role in suppressing endothelial cell proliferation, inhibition of protease activities in tissues undergoing remodeling of the extracellular matrix, and possessing erythroid-potentiating activities [63–66]. High expression levels of TIMP-2 in breast cancer are associated with poor prognosis [67], whereas low expressions of TIMP-2 in lung cancer are correlated with poor prognosis [68]. In addition, high expression levels of TIMP-2 in tumor tissues and serum of liver cancer patients were associated with decreased metastases [69]. However, the roles of TIMP-2 in CRC prognosis and CRC drug resistance have not been elucidated.

We found that CRC patients with elevated TIMP-2 levels exhibited poor overall survival (OS), disease-free survival (DFS) and disease outcomes. These findings were a confirmation of preliminary clinical and 5-Fu-resistant PDX model results that showed a high expression of TIMP-2 in drug-resistant CRC. Therefore, TIMP-2 is a potential marker for 5-Fu drug resistance in CRC patients. Since elevated TIMP-2 levels inform the prognosis of 5-Fu-resistant CRC patients, it is important to evaluate TIMP-2 levels in blood during chemotherapy to assess 5-Fu resistance as early as possible. Elevated TIMP-2 expression levels are accompanied by changes in patient's 5-Fu resistance status. Consequently, doctors can act appropriately to prevent tumor progression. However, studies should elucidate on the relationship between TIMP-2 expression levels and clinical patient characteristics to ascertain these findings.

Our cellular experiments confirmed the ability of TIMP-2 to cause resistance in CRC cells. Li et al. reported a new autocrine cytokine expression following drug resistance in leukemia [70]. Therefore, we aimed at determining whether TIMP-2 induces 5-Fu resistance through this mechanism. 5-Fu sensitive cells co-cultured with 5-Fu resistant cells with a survival advantage were used to determine the cytokines endowing CRC with 5-Fu resistance. Effects of recombinant TIMP-2 treatment on CRC cells revealed that secreted TIMP-2 acts as an autocrine factor to induce 5-Fu resistance. Inhibition of TIMP-2 by neutralization antibodies or siRNA reversed drug resistance in 5-Fu resistant cells. Therefore, up-regulation, down-regulation and rescue experiments proved that an autocrine mechanism is involved in TIMP-2 induced colorectal cancer cell resistance to 5-Fu. These findings elucidate on the role of anti-TIMP-2 antibody in preventing CRC patients from acquiring resistance to 5-Fu drugs during treatment. Moreover, serum TIMP-2 levels in CRC patients are potential biomarkers for evaluating potential resistance of patients to 5-Fu treatment.

A small-molecule inhibitor (U0126) has been shown to target key proteins in the ERK/MAPK signaling pathway [44, 45]. We found that TIMP-2 mediates 5-Fu resistance through the ERK/MAPK signaling pathway in CRC cells. Targeting the ERK/MAPK signaling pathway can re-sensitize 5-Fu resistant CRC cells to 5-Fu. From our results, we infer that U0126 can efficiently switch 5-Fu-resistant CRC cells to 5-Fu sensitive CRC cells due to its ability to inhibit the ERK/MAPK signaling pathway and to block the TIMP-2 autocrine mechanism involved in 5-Fu resistance.

Therefore, combined use of an agent targeting TIMP-2 and 5-Fu has the potential for preventing or treating CRC resistance to 5-Fu in CRC patients. Alternatively, small molecule inhibitors that target the ERK/MAPK signaling pathway, such as U0126, can effectively cut off the pathway, thereby increasing sensitivity of colorectal tumors to 5-Fu. However, studies involving animal experiments and clinical trials should be performed to ascertain these findings.

## CONCLUSIONS

TIMP-2 is overexpressed in CRC patients, which promotes drug resistance to 5-Fu through the EPK/MAPK signaling pathway. This elevation is indicative of poor disease prognosis. CRC resistance to 5-Fu can be regulated by inhibition of TIMP-2 or ERK/MAPK signaling pathway. Finally, combined administration of TIMP-2 or ERK/MAPK signaling pathway inhibitors and 5-Fu is a promising chemotherapeutic option for the treatment of first time CRC patients as well as relapsed CRC patients previously treated using 5-Fu-based chemotherapy.
